# Nursing in a different world: Remote area nursing as a specialist–generalist practice area

**DOI:** 10.1111/ajr.12899

**Published:** 2022-06-30

**Authors:** Kylie McCullough, Sara Bayes, Lisa Whitehead, Anne Williams, Vicki Cope

**Affiliations:** ^1^ School of Nursing and Midwifery Edith Cowan University Joondalup WA Australia; ^2^ Australian Catholic University and Edith Cowan University Joondalup WA Australia; ^3^ Edith Cowan University Joondalup WA Australia; ^4^ Murdoch University Perth WA Australia

**Keywords:** advanced practice nursing, health services, indigenous, nurse practitioners, primary health care, rural nursing

## Abstract

**Objective:**

Remote area nurses provide primary health care services to isolated communities across Australia. They manage acute health issues, chronic illness, health promotion and emergency responses. This article discusses why their generalist scope of practice should be formally recognised as a specialist nursing practice area.

**Design:**

Constructivist grounded theory, using telephone interviews (*n* = 24) with registered nurses and nurse practitioners.

**Setting:**

Primary health care clinics, in communities of 150–1500 residents across Australia.

**Participants:**

A total of 24 nurses participated in this study.

**Results:**

Nurses' perceived their clinical knowledge and skill as insufficient for the advanced, generalist, scope of practice in the remote context, especially when working alone. Experience in other settings was inadequate preparation for working in remote areas. Knowledge and skill developed on the job, with formal learning, such as nurse practitioner studies, extending the individual nurse's scope of practice to meet the expectations of the role, including health promotion.

**Conclusion:**

Remote area nursing requires different knowledge and skills from those found in any other nursing practice setting. This study supports the claim that remote area nursing is a specialist–generalist role and presents a compelling case for further examination of the generalist education and support needs of these nurses. Combined with multidisciplinary collaboration, developing clinical knowledge and skill across the primary health care spectrum increased the availability of health resources and subsequently improved access to care for remote communities. Further research is required to articulate the contemporary scope of practice of remote area nurses to differentiate their role from that of nurse practitioners.

**What is already known on the subject:**

- Remote area nurses require a broad range of clinical and primary health care skills, such as health promotion and community engagement, due to a lack of access to other health professionals
- Remote area nurses report concerns about a lack of support for skill development

**What this paper adds:**

- Specialised education and support that includes acute care, public health and health promotion for nurses working in remote areas is essential for the provision of safe and quality care to remote populations
- Recognition of the generalist nature of primary health care nursing practice in remote areas as a distinct nursing speciality would provide clearer education and career pathways particularly if nurse practitioner endorsement was encouraged

## 1 BACKGROUND

Nurses working in small remote communities in Australia are commonly referred to as remote area nurses (RANs). This informal term reflects a specialist field of nursing practice where the lack of health resources and the nature of the remote setting require a wide range of clinical and primary health care skills and knowledge applied in an environment of geographical and professional isolation.^1^ While access to health practitioners decreases in relation to remoteness, the number of FTE registered nurses per 100 000 people is higher in remote and very remote areas than in metropolitan or rural areas.^2^ However, this statistic is deceptive as it does not account for the dispersion of the population across vast areas and the subsequent travel distance required to access these nursing services. Furthermore, the higher number of nurses per population reflects the need for nurses to be substitutes for the lack of other health practitioners such as General Practitioners, Paramedics and Allied health.

Nursing practice in the remote setting involves a component of General Practitioner substitution, and at times extends beyond the normally expected scope of nursing practice to include roles normally provided by allied health practitioners such as X‐rays, ambulance response and medication dispensing.^3^, ^4^ Furthermore, nursing practice includes health promotion and public health activities as well as non‐clinical tasks such as cleaning, property and vehicle maintenance, reception and administration duties and even animal health.^4^

Approximately 80% of remote area nurses work in a cross‐cultural environment which requires nurses to adapt to different languages, social structures and traditions as well as differences in the burden and presentation of disease.^5^ Cramer (^6^ p.201) describes remote area nursing as being ‘…completely different from nursing as it is generally practiced in other settings’ largely due to: the lack of boundaries to practice, a medical rather than nursing focus (which includes a doctor substitute role), social and professional isolation and unrealistic expectations of communities and employers.

There is a paucity of research describing the practice of remote area nurses despite the specialised context of practice. However, issues such as retention and turnover of staff, workplace safety, employment conditions and stress in remote nursing populations have been explored.^7^, ^8^ Although not directly related to nursing practice, these issues are highly relevant where increased retention of health care workers is associated with advanced clinical skills and better continuity of care.^9^ Conversely, high turnover of healthcare workers results in a loss of resources (or ‘corporate knowledge’) particularly in small communities,^10^ inconsistencies in treatment and advice given, lack of follow‐up and a high rate of expensive patient transfers to regional hospitals.^11^ Unfortunately, turnover rates for remote health professionals, especially nurses, are extremely high^9^ with the shortage expected to worsen.^8^ For example, the Northern Territory saw an increase in the use of short‐term agency and Fly‐in'Fly‐out (FIFO)/Drive‐in‐drive‐out (DIDO) workers between 2004 and 2015 in response to the high turnover of nurses in remote clinics.^12^, ^13^ In time, studies will report on the impact of COVID‐19 on workforce supply.^12^ Anecdotal reports indicate that interstate and international restrictions have limited the supply of nurses, and some remote jurisdictions have been unable to safely staff remote clinics resulting in clinic closures.

### 1.1 Remote area nursing as a specialist–generalist nursing role

Most nurses working in remote areas are registered nurses,^14^ which means that they have completed an approved undergraduate program, are registered with the Nursing and Midwifery Board of Australia (NMBA) and are required to adhere to professional practice standards and conduct. The dual registration as nurse and midwife makes quantifying access to midwifery care challenging; however, the number of nurses in remote areas with midwifery qualifications is reported to be low.^8^ This means some communities will not have access to registered midwives on site thereby creating reproductive inequalities for women in remote areas.^15^ In the absence of midwives or General Practitioners, the registered nurse's scope of practice may include a degree of antenatal and postnatal care.

Pre‐registration nursing curricula do not prepare nurses for practice in remote areas^16^ and mostly focus on preparing graduates for acute specialist roles rather than primary health care positions.^17^ While a comprehensive orientation program, post‐registration education opportunities and guidance from clinical practice manuals^18^ can assist the new remote area nurses in their role as specialist–generalists,^3^ there is a gap in knowledge regarding the needs of nurses who become ‘specialist– generalists’ in the remote setting.

Some remote area nurses continue their studies and become nurse practitioners with a broader scope of practice than registered nurses. The advanced and extended practice of nurses in remote areas has been an argument for the introduction of nurse practitioners in Canada and Australia.^3^, ^19^, ^20^, ^21^ The practice followed recognition that remote area nurses were often working outside of their usual scope of practice and legal requirements of registered nurses, particularly in relation to the use of medicines.^22^ Banner et al.^23^ also note a ‘double‐standard’ that has allowed registered nurses to function in an advanced and extended role in rural, remote and Indigenous communities when they would not be permitted to do so in an urban setting. This anomaly in nursing practice is criticised as encroaching on the rights of people living in remote areas and adding to the inequality in health status, particularly in Indigenous communities.^19^ In Australia, there are very few nurse practitioners working in remote areas,^24^ with only 72 full‐time equivalent positions from a total pool of 1477 nurse practitioners Australia wide working in remote or very remote areas.^25^ Research regarding the work of nurse practitioners and how this differs from remote area nurses as specialist–generalists are not reported in the academic literature.

There is a need for a better understanding of the different roles of nurses within the remote setting so that their contributions to health outcomes can be recognised. Furthermore, a deeper understanding of what it means to be a specialist–generalist practitioner is of relevance to primary health care settings more broadly where nurses are providing comprehensive care to communities.

**FIGURE 1:**
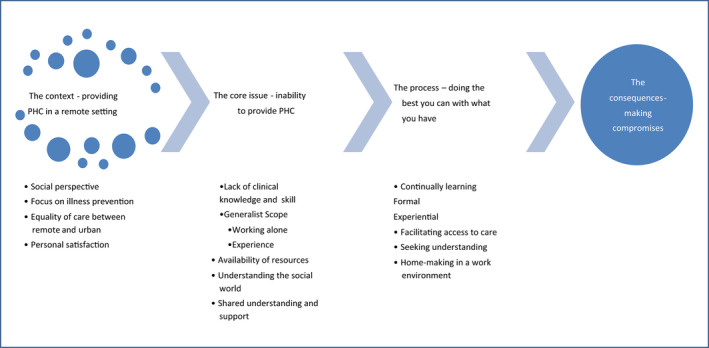
The substantive theory: Making compromises to provide primary health care in a remote setting

In this study, nursing practice in remote areas is explored and the argument presented that remote area nursing practice is an advanced and specialised nursing role. The findings reported herein form part of a larger study of the PRIMARY HEALTH CARE role of nurses in remote Australian settings,^26^, ^27^, ^28^ which aimed to describe the actions and interactions used by nurses, the contexts and conditions and the factors which enhanced or inhibited the delivery of primary health care in the remote setting. An overview of the theoretical framework developed in the larger study, with the findings of this study highlighted in bold text, is presented in Figure 1 below:

## 2 DESIGN

Grounded theory methodology was used to describe and explain from the perspective of nurses the actions and interactions used to deliver primary health care in remote communities. Grounded theory methodology is particularly useful when seeking to explain as well as describe behaviour. It allows the development of an abstract framework of understanding which is often applicable to many areas of practice even when the setting is context specific.^29^ Further methodological detail can be found in the original study publication.^28^

### 2.1 Setting and participants

The setting for this study was remote primary health care clinics without inpatient facilities that served populations ranging from 150 people to around 1500 residents. Twenty‐four nurses participated in this study. Thirteen of them were nurse practitioners and the remaining were registered nurses. Eleven participants were concurrently registered as midwives and 16 held Master's degrees. Half of the participants had more than 10 years' experience working in remote areas with a range of 3 months to 15 years. Four were males and 20 were females. Collectively they had worked across Australia in the Northern Territory, Western Australia, Queensland and the Indian Ocean Territories. A substantive theory was inductively derived from telephone interviews (45–120 min duration) with remote area nurses across Australia between 2014 and 2017 as well as an expert reference group (*n* = 4) and informal feedback from remote area nurses at two industry conferences. Participants were recruited via the snowball method commencing with members of a nurse practitioner's interest group.

### 2.2 Data collection and analysis

Data were analysed through constant comparison and theoretical sampling according to the principles described by Charmaz.^29^ Theoretical sensitivity arose from the chief investigators' experience as a remote area nurse and reflexivity was enhanced through a process of self‐reflection undertaken by the researcher in order to identify bias and articulate the effect the researcher has on data collection and analysis.^30^ Data were analysed in relation to symbolic interactionism which is a theoretical framework that guides an in‐depth understanding of the social processes that occur in nursing practice.^31^ Trustworthiness was assessed according to Charmaz's^29^ criteria of resonance, originality, usefulness and credibility during the presentation of emergent findings at industry conferences, the contribution of the expert reference group, academic oversight from study supervisors and the subsequent publication and citation of the original theory paper by peer‐reviewed journals.

### 2.3 Ethical considerations

Ethics approval to conduct the study was provided by Edith Cowan University Human Research Ethics Committee, protocol number 10810. COREQ reporting guidelines were followed^32^ and trustworthiness met in line with Charmaz^29^ expectations. Participant identities were anonymised during verbatim transcription of the interview audio recordings. Participants were encouraged to access emotional support if needed via the CRANAplus Bush Support Service (https://crana.org.au/mental‐health‐wellbeing/call‐1800‐805‐391), but no reports of distress as a result of participating in this project were made.

## 3 RESULTS

The substantive theory labelled ‘*Making compromises to provide Primary Health Care*’ (Figure 1) explained how nurses provide primary health care in the remote setting.^28^ In this theory, primary health care was a social model of care based on a social justice paradigm that went beyond attending to acute presentations to include health promotion and community development. The main problem for these nurses was their *inability to provide care* in line with their philosophy of primary health care. They managed this conflict by using a process in which *they did the best they could with what they had*, which led to the outcome of *making compromises* to provide primary health care. Four conditions were found to influence the strategies they used to manage the main problem: *lack of understanding of the social world, lack of resources, lack of shared understanding and support* and, notably the focus of this study, *a lack of clinical knowledge and skill*. Within this condition, working alone and level of personal experience impacted their individual scope of practice.

### 3.1 Lack of knowledge and skill that reflects the generalist scope of practice

Participants frequently expressed a lack of clinical knowledge and skill as a fundamental variable in their ability to provide primary health care. The lack of knowledge specific to a generalist role, such as a community primary health service, created a discrepancy between the scope of practice needed and their competence and confidence in providing that level of care. This discrepancy was frequently expressed as ‘difference’. They described feeling as though their knowledge and skills from previous nursing roles were irrelevant as the remote context was so different from what they had previously experienced. NP12 described it thus: ‘*All the traditional boundaries and knowledge base; everything that I had gone with before I had to throw that out the window and start again’*. The lack of skills was interpreted as impacting negatively on the quality of care provided, as NP14 indicated: *‘the care of the patients is impacted a lot by the lack of skills’*.

The lack of knowledge was in part because the nurses did not have prior experience with the health needs of people in the remote setting: ‘*You will see things here that you will never see anywhere else’ (NP12)*. The knowledge deficit appeared to be particularly relevant for nurses who primarily worked with Aboriginal or Torres Strait Islander people:

…[I] wanted something extra [in my nursing studies] about the health issues and the co‐morbidities of Aboriginal people and the issues in treating from that point of view, …because it is different, there's no doubt that it is different. (NP12)

Nurses described needing to have a generalist scope of practice and recognised that this was different from the specialised roles nurses undertake in urban settings. NP10 described how *‘…the varied nature of the role; so it was a lot broader than I guess what I'd experienced before’* and that *‘…coming from a much more specific, targeted health service that was a bit of a challenge’*. In order to attend to such a variety of individual and community health needs, generalist knowledge was described as, ‘*…you need to have some information about a broad range of subjects; and if you don't know you have to know where to go to get it’ (RAN4)*. Of note, nurses new to remote areas described having a limited understanding of health promotion, screening and management of chronic disease:

… I knew that there was a hole in my education … the chronic disease management, it was a whole new thing …and doing child health checks…that sort of stuff, I just didn't know anything about it. (NP8)

Expertise in the remote context included a level of clinical assessment; reasoning and treatment knowledge not often required of nurses in other contexts. RAN6, for example, said;

… when you are working in ED, the final decisions aren't really lying with you. The assessment skills … only go to a certain point and then the doctor takes over with any treatment…as a RAN, you take on a wider scope, you are certainly doing a greater number of assessments; …greater responsibility and decisions – even though you are using clinical protocols you have to interpret those for how you are using them. (RAN6)

### 3.2 Working alone

Another point of difference between urban nursing contexts and remote setting was the requirement to provide a 24‐h service to cover for emergencies. In practice, this meant that generalist nurses needed to deal with any potential emergency on site, including retrieving patients from outside the clinic setting or doing whatever was necessary in preparation for retrieval by air or road. After hours, nurses generally lacked access to other health professionals on site and, as NP4 said, *‘… RANs have a very diverse skill range because they have to be independent working away from resources*’. In the absence of paramedics or access to the resources of a tertiary hospital, nurses working in remote areas were required to attend roadside retrievals as well as provide the clinical care required to triage, stabilise and continue treatment of the patient until discharge. As RAN1 explains it,

…all of a sudden you weren't just being a nurse you were being a paramedic …police officer and whatever else comes with all that roadside stuff and so that was crazy and I had hardly cannulated anyone and all of a sudden we were cannulating people who were dying …it was huge. (RAN1)

Working autonomously led to greater responsibility and a need to provide care for a wide range of clinical presentations:

…I don't think I could get this kind of experience anywhere else other than working remote …the acuity and the mix, you don't have anyone to hand over to, there's no one to come in and say ‘ok, we'll manage this patient from here on in’ …it's a huge challenge, it's a much wider scope of practice than you would normally get anywhere else …and I love it. (NP12)

Nurses in this study described feeling *scared (RAN5), nervous (RAN4) and frightened (NP1)* at times when their skills and experience did not prepare them for the situation at hand. Concern about the scope of practice was frequently expressed by nurses in this study, as they were the only ones available with health knowledge and access to resources within the community and so needed to do things they had never experienced before:

…the scope of practice remote area nurses are expected to have is huge and …it's scary sometimes …you're faced with something that you have little or no training in basically, but …if you don't do it or give it a go, is anyone else going to be able to? or do we try and evac[uate] this person at the cost of many thousands of dollars and inconvenience to them? …the questions are pretty big sometimes. (RAN1)

The ‘big questions’ referred to in the previous quote demonstrate the complicated decision‐making process required by generalist nurses and the vast array of other factors that needed to be considered when decision‐making, including the cost to the health system (resource utilisation) and impact on the patient.

### 3.3 Experience: ‘it takes time to know this stuff’

Increasing their own knowledge and skill increased the health resources available to that community. In this study, nurses engaged in formal and experiential learning activities. Opportunities to learn and increase knowledge and skill occurred over time and exposure to a variety of clinical situations and through interactions with the community, as a result, they were required to, *‘learn on the job’ (NP1)*. Learning generalist skills on the job was interpreted as being more than just clinical skills:

To work remote, you need your clinical skills, but you also need a lot of other skills that aren't clinically based for sure …so who do you go to in the community? It has nothing to do with being a nurse but rather how to operate in those remote communities; you need to know the logistics of the place. (NP1)

Finding opportunities to participate in learning was a response to a deficit in knowledge that came from an internal motivation of wanting to provide the best care. For instance, RAN6 said ‘… [I'm becoming a Nurse Practitioner because] …*I wanted to know more. I wanted to be able to do what I am doing but better … I wanted to feel that people trusted what I said’*.

However, professional development and learning opportunities were not always freely available and the process of learning in a remote setting was difficult. NP7 provided an example of how she addressed this challenging issue: ‘*…I do a lot of online stuff and I tried to do some professional development every year …but I try to get involved in anything that CRANA's* [professional organisation representing remote health] *throwing out and …but yeah it's hard, really hard’ (NP7)*. Several post‐graduate courses were discussed by participants, including a transition program supported by government employers. Some shared their learning pathway in which one qualification led to another:

I came with no remote experience so for me I started off …by doing the Grad[uate] Cert[ificate] and then I went on and did the Masters in Remote Health which led to NP so from there I kind of fell into it a little bit but to me it was a natural way it evolved as I was already working in extended practice. (NP3)

Engagement in formal learning in order to build knowledge resources was described as one effective way of reducing the lack of knowledge and skill. It was evident, however, that experiential learning was highly valued in the remote setting. Learning on the job was a challenging process described as being, ‘…*thrown in the deep end*’ (NP4). Although stressful, these situations were valuable learning opportunities when people were available to teach and support, particularly when providing care in a cross‐cultural setting: ‘*… people corrected me and educated me… a lot of it is role modelling and reading and watching’ (NP4)*.

It was clear that continually learning was a valuable strategy for building knowledge and skill. Increasing the knowledge resources of the individual nurse, in turn, increased the health resources available to the community.

### 3.4 Nurse practitioners: the ultimate specialist–generalists?

Some of the participants in this study were nurse practitioners. To be endorsed as a nurse practitioner with remote scope of practice, they needed to have at least 5‐year experience working within a remote setting. They claimed to have a different scope of practice to registered nurses and described their practice as providing ‘…*a more comprehensive assessment’ (NP11)* because they ‘*… think a lot more broadly and to incorporate a lot of other factors into your assessment. Your assessment skills and thinking and reasoning is a lot greater’ (NP1)*. Furthermore, their scope of practice extends beyond clinical knowledge and skill:

…the value of a nurse practitioner in a remote area is actually their ability to connect teams together, their ability to think on their feet and use the resources they've got, their decision‐making in situations that they are not familiar with or that things are complicated; their education ability… those things rather than clinical assessments, treatments. (NP2)

It was apparent that nurse practitioners considered themselves practice experts within a remote primary health care setting, however, there were very few nurses in these roles. This anomaly was explained to be due to a lack of designated nurse practitioner positions,

a lot of remote nurses… have been working at the level of a nurse practitioner …and got their qualification and endorsement …but then there's still not the positions… to flow into and so we carry on just being a remote area nurse. (NP1)

While the lack of clinical knowledge and skill was identified as having a significant impact on nurses' ability to provide primary health care services in remote areas, continually learning was a strategy that increased the scope of practice of nurses and subsequently provided access to a greater range and higher quality of patient care. Conversely, the high rate of staff turnover was seen to diminish the availability of specialist–generalists within the remote setting because they never really came to understand the community context over time or develop skills through experiential learning:

…in [community] they've got six nurses and they probably turn over every 2 weeks, 3 weeks, 4 weeks they turn over. You know you get that cycle going for 4 years and you see a lot of nurses coming and going, the same with doctors and stuff like that and there's very little continuity of care. (RAN2)

Transient staff were described as avoiding health promotion activities due to a lack of knowledge about primary health care programs, and management of chronic disease seemed to be particularly adversely affected by high staff turnover, as RAN4 explained: ‘*…chronic disease is becoming more and more complex and then when you've got a high turnover of staff, …well, you know it just doesn't get done properly’ (RAN4)*. In addition to a lack of continuity, a lack of awareness of available resources impacted the provision of care:

There are some great resources available up here and people don't end up getting referred or knowing about them because the staff themselves don't know …. You can't learn all that stuff in 6–8 weeks. It takes [time] to know all this stuff. (NP1)

## 4 DISCUSSION

The results presented here focus on the scope of practice as a barrier or facilitator to nurses' ability to provide primary health care in the remote setting as proposed by McCullough et al.^28^ In this study, remote area nurses described their work as different form other nursing roles. We suggest that this difference from other nursing roles supports the classification of remote area nursing as a specialty practice area. They also described needing a generalist scope of practice to provide care to a whole community inclusive of acute and chronic conditions, as well as health promotion activities.

To meet the needs of the community, nurses described a requirement to work autonomously, particularly outside of business hours, and to be able to provide culturally safe care in a cross‐cultural environment. Nurses recognised a requirement for experiential and formal learning opportunities in order to develop the knowledge and skills required for a broad scope of practice, and nurse practitioners were described as best placed to provide generalist care. These factors formed part of the process of doing the best they could, in order to provide primary health care. In relation to the substantive theory (Figure 1), the presence or absence of clinical knowledge and skill is predicted to impact the nurse's ability to provide primary health care. Future research designed to measure the relationship between nursing knowledge and skill with patient outcomes is needed.

### 4.1 Generalist knowledge and skill as a specialty practice area

A perceived lack of clinical knowledge and broader generalist skill, particularly when working alone, has also been described by several authors.^6^, ^7^, ^33^, ^34^ Notably, in her thesis on the experience of locum nurses, Becker^33^ described nurses entering the remote setting as ‘…urban‐based professionals with urban‐based education’ (^33^ p.167). Her findings described confusion about the scope of practice and concern about the level of decision‐making that was required. In this current study, participants observed other nurses with little or no experience in this setting as having a narrow acute care focus that reflected their specialist urban knowledge and skill rather than a broad generalist scope of primary health care that included health promotion and public health.^28^ Lundberg et al.^35^ in their study of nurses working on a remote island in Finland also reported that the nature of nursing consultations was extremely varied and focused on the disease rather than broader social determinants of health. Acknowledging the specific skills and knowledge within the remote primary health care setting that are needed for generalist practice is the first step in creating a workforce that can provide services that meet community health needs.

Given the need for specialist–generalist skills and knowledge that are different from that attained by nurses working in hospital environments, it is not surprising that participants in this study described feeling anxious when they were required to manage clinical situations or perform tasks they have not been prepared for, including health promotion and community engagement. This study highlights the essential requirement for on‐the‐job learning in addition to formal learning. Ashley et al.^36^ described registered nurses who transitioned from acute care to primary health care as commonly reporting difficulties with adjusting to the new practice setting. Almost half of their participants reported feeling isolated, unsupported or overwhelmed with the transition process. Providing supervision and support during the transition from acute care nurse to remote primary health care nurse is vital in building clinical knowledge and skill appropriate to this setting.

### 4.2 Scope of practice

Nurses in this study described frequently questioning whether a particular situation was within their scope of practice. This indicates confusion about the legal standing of some nursing activities in the remote setting, particularly in situations where nurses are working alone and have to attempt a task where they have not been formally instructed or assessed as competent. Nursing standards and codes of conduct^37^, ^38^ do not specifically define what tasks nurses can and cannot do. Furthermore, a myriad of other legislation impacts nursing practice, including the Poisons and Dangerous Substances Acts; Privacy and Confidentiality Acts; and Occupational Health and Safety Acts.^39^, ^40^ Therefore, context plays an important part, and a judgement of negligence requires analysis of the actions of the nurse as compared to what would be the expected actions by a ‘jury’ of peers. Kerridge and associates describe it thus:

…A health professional does not incur liability if it can be established that he or she acted in a manner in Australia by peer professional opinion as competent professional practice^41^

Kerridge et al^41^ also discuss how courts consider the context of an individual case ‘…the standard of care owed by a health professional will be different in a remote community compared with a modern metropolitan hospital’. This statement does not excuse poor care but indicates the importance of understanding the impact of context on practice and the need to formally recognise that nursing in remote areas is a specialty practice area.

Therefore, the context and limitations of remote, generalist and primary health care nursing practice should be recognised as requiring advanced and specialised scope of practice. This includes the need for cultural competency and specific health knowledge regarding Aboriginal and Torres Strait Islander people's health needs.^42^, ^43^

However, just because there is a need for a particular skill or knowledge does not automatically mean that an individual nurse is competent and confident to provide that service. The term ‘Remote Area Nurse’ is currently adopted by nurses who work within the remote setting regardless of how long they have worked there, or whether they have any formal qualifications relating to the remote context. In response to this situation, a professionally credentialed Remote Area Nurse designation has been promoted and administered by CRANAplus, the peak professional body for the remote and isolated health workforce of Australia, in order to formally recognise nurses who can demonstrate that they are practicing within the remote standards of practice framework.^44^ In the future, as more nurses undertake the process to formally adopt the term remote area nurse, it may be easier to describe remote nursing practice as an advanced specialty because research could differentiate among a registered nurse working in a remote setting, a credentialled remote area nurse and a nurse practitioner when evaluating the impact of nurses on patient health outcomes. Nurses in the remote setting should be encouraged and supported to maintain a contemporary portfolio of practice and complete credentialling requirements to demonstrate their own scope of practice.

Nurse practitioners are expert nurses and in Australia will have successfully completed postgraduate Masters' degree, a minimum of 5 years' experience in their area of specialty and undergone a process of independent review in order to gain endorsement. Their scope of practice includes the ability to prescribe medications and order radiology and pathology tests. It is a clinically focused role and includes research, education and leadership in clinical care.^37^ In Canada, nurse practitioners are recognised as an essential component of primary health care reform.^19^, ^45^ Mills et al.^46^ also claim that nurse practitioners in rural and remote areas have the potential to have a positive impact on health care and that health authorities should be actively creating nurse practitioner positions within their organisations. Carryer et al.^47^ also propose that nurse practitioners in primary health care could be the catalyst for transforming health delivery in New Zealand, as a way of better meeting the rising need for health services as a result of ageing populations, chronic disease and increasing health inequality.

### 4.3 Developing expertise

In this study, ‘experience’, was informally assessed by the participants in terms of length of time spent in the remote context. Experience was a term used interchangeably with ‘expert’ by the participants. This was due to the largely experiential, on‐the‐job learning that occurred and the importance of understanding the social world and context of the community. An earlier study suggested that it took around 4 years to become an experienced remote area nurse.^48^ The development process of gaining nursing expertise can be considered in light of Benner's theory: Novice to Expert^49^ in which expertise is described as mature practical knowledge of the patient population within the clinical world. Benner's theory is unidirectional and posits the development of expertise as progressive, however, the current study identified a perceived regression in nursing expertise when participants described ‘…*starting again*’ (NP12), as the context was so different from other nursing specialty areas of practice.

This study contributes to understanding the educational needs of nurses transitioning from an urban acute setting to a remote primary health care setting. To that end, postgraduate education has been found to enhance the ability of nurses to undertake health promotion activities, particularly in response to chronic disease, women's health and education.^50^ However, Whiteing et al.^34^ found that practical experience was perceived as more valuable by registered nurses compared to formal learning opportunities. Muirhead et al.^4^ highlight the importance of flexible learning opportunities which respond to individual learning needs and setting of the nurse. Furthermore, McFarlane et al.^51^ describe the need for skill development in health promotion for remote nurses that has a particular emphasis on the needs of Aboriginal and Torres Strait Islander communities. Further research is needed to understand the health promotion education needs of remote area nurses. The current study indicates that there is an additional need to understand the advanced practice nature of developing specialist–generalist knowledge and skill within a primary health care setting. The Australian College of Nursing^52^ support the training of generalists as opposed to specialists to enhance the provision of primary health care in rural and remote communities.

### 4.4 Nursing knowledge as a community resource

In this study, nurses who became specialised in the remote primary health care context described themselves as better able to provide primary health care. This was primarily associated with the belief that as nurses extended their scope of practice to become generalists, they were able to expand the range of health services to the community. Indeed, The National Rural Health Alliance Inc.^53^ recognises the importance of clinical, cultural and remote contextual experience and the contribution this makes to improving health. Muirhead et al.^4^ also suggest that the quality of care provided is dependent on the experience and expertise of nurses.

Farmer et al.^54^ proposed a theory of the contribution of health services to the social capital and sustainability of rural communities. They identified contributions made by individual health professionals to social capital in terms of sharing personal knowledge, skills and qualifications, contribution to the social aspects of a community through participation and informal health and social care, and economic contributions from personal consumption of goods and services. This theory resonates with the current study, in which nursing expertise has been positioned as knowledge capital and its value as a community resource confirmed. Therefore, we suggest that supporting the generalist education and skill development of nurses would aid in overcoming some of the resource limitations inherent in the remote setting.

## 5 STUDY LIMITATIONS

The generalisability of the study findings is limited by the small sample size. The experiences of the participants were not differentiated between those gained when working as a registered nurse or nurse practitioner but rather reflected their experiences of working in the remote setting which included working as a registered nurse in the remote setting before transitioning to a nurse practitioner role. Furthermore, data on the nature of postgraduate qualifications (award, specialty and endorsement) were not collected.

This limitation also supports a finding presented in this study as to the lack of clarity in the scope of practice between specialist–generalist remote registered nurses and nurse practitioners. Studies that aim to describe nursing practice at a single time point would better enable comparison between different levels of expertise. Further research with a larger sample from a wider variety of settings, including remote areas in other countries, would further enhance and develop this substantive theory.

## 6 CONCLUSION

This study focuses on one key finding of a broader study that developed a theory of primary health care nursing practice in the remote setting. The lack of generalist clinical knowledge and skill, which includes the broad spectrum of primary health care activities, was significant in that providing nursing care in remote settings required specialised general knowledge and skills compared to those found in any other nursing practice setting. Nurses were found to feel unprepared educationally and clinically for remote environments even if they had many years of nursing experience, and they needed support and time to grow and adapt to this different world. Learning occurred on the job and through the attainment of formal post‐graduate qualifications. Combined with multidisciplinary collaboration, developing clinical knowledge and skill increased the availability of community resources and improved access to care for remote communities. This study contributes to the discussion regarding remote area nursing as a specialised field of nursing with a generalist scope of practice. Further research is required to articulate the current, contemporary scope of practice of remote area nurses which will inform the clinical decision‐making of nurses and the educational support necessary for nurses to provide safe, quality care. Furthermore, research that describes the work of nurse practitioners in remote areas will aid in understanding the advanced and specialist nature of this specialist–generalist nursing role.

## AUTHOR CONTRIBUTIONS

KMM: conceptualization; data curation; formal analysis; funding acquisition; investigation; methodology; project administration; writing – original draft; writing – review and editing. AW: conceptualization; data curation; formal analysis; methodology; supervision; writing – review and editing. LW: formal analysis; methodology; supervision; writing – review and editing. SB: formal analysis; methodology; supervision; writing – review and editing. VC: conceptualization; formal analysis; supervision; writing – review and editing.

## CONFLICT OF INTEREST

Results of this study have been published in part elsewhere (references below). The findings presented in this study have not been published other than in the first authors’ entire thesis which is available through the university repository. These citations have been removed from the manuscript during the review process to maintain blind peer review.

Other papers from this study

McCullough, K., Whitehead, L., Bayes, S., Williams, A., & Cope, V. (2020). The delivery of Primary Health Care in remote communities: A Grounded Theory study of the perspective of nurses. *International Journal of Nursing Studies*, *102*, 103 474. doi:j.ijnurstu.2019.103474

McCullough, K., Bayes, S., Whitehead, L., Williams, A., & Cope, V. (2021). We say we are doing primary health care but we are not: Remote area nurses' perspectives on the challenges of providing primary health care services. *Collegian*.

McCullough, K., Whitehead, L., Bayes, S., & Schultz, R. (2021). Remote area nursing: best practice or paternalism in action? The importance of consumer perspectives on primary health care nursing practice in remote communities. *Australian Journal of Primary Health*, *27* (1), 62–66.

## ETHICAL APPROVAL

Ethics approval to conduct the study was provided by Edith Cowan University Human Research Ethics Committee, protocol number 10810.
